# Supplementary material for: The unequal functional redundancy of Arabidopsis INCURVATA11 and CUPULIFORMIS2 is not dependent on genetic background
Source: Front Plant Sci. 2023 Nov 15;14:1239093. doi: 10.3389/fpls.2023.1239093 (PMC10684699; doi:10.3389/fpls.2023.1239093)
Supplement: Supplementary file 1 [file DataSheet_1.pdf]

The unequal functional redundancy of the Arabidopsis *INCURVATA11* and *CUPULIFORMIS2* genes is not dependent on genetic background

Riad Nadi, Lucía Juan-Vicente, Eduardo Mateo-Bonmatí*, and José Luis Micol

Instituto de Bioingeniería, Universidad Miguel Hernández, Campus de Elche, 03202 Elche, Spain

*Current adress: Centro de Biotecnología y Genómica de Plantas (CBGP), Universidad Politécnica de Madrid (UPM) – Instituto Nacional de Investigación y Tecnología Agraria y Alimentaria (INIA)/CSIC, 28223 Pozuelo de Alarcón, Madrid, Spain.

Supplementary Figures and Tables

Supplementary Material not included in this file:

Supplementary Table S2

A

Features of sgRNA and its target

sgRNA name: ICU11_sgRNA1

sgRNA sequence: GCGAGGCAAGATTGAAGCTTCGG

Target location: Chr1:8127046. AT1G22950, first exon

Target strand: complementary

sgRNA efficiency: 49.72%

sgRNA predicted off-targets: MM(0):0, MM(1):0, MM(2):0, MM(3):0, MM(4):2

B

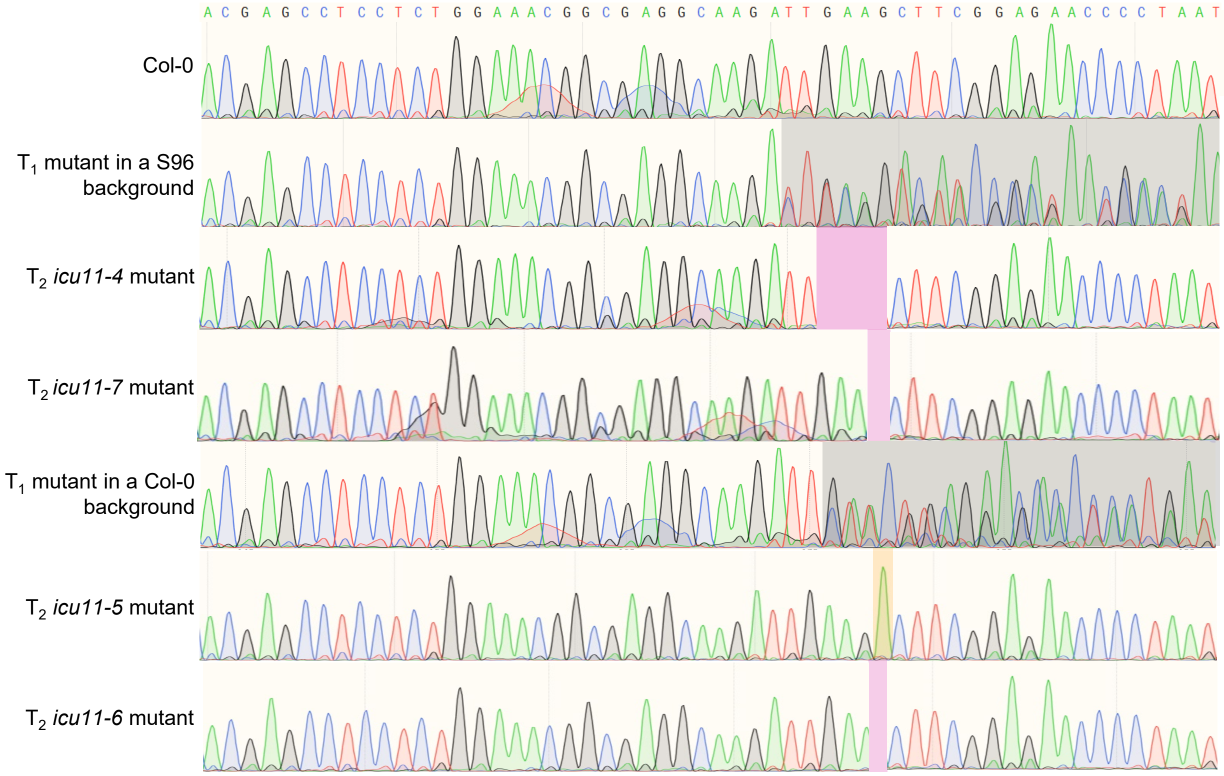

C

```
ICU11-4      1 MCNQTPLRSMALDSSGKQPEQQQQQQPRASSGNGEARFFGEPLMKNMSLRTMKIYLWTIV
ICU11-6      1 MCNQTPLRSMALDSSGKQPEQQQQQQPRASSGNGEARLNFGEPLMKNMSLRTMKIYLWTI
ICU11-7      1 MCNQTPLRSMALDSSGKQPEQQQQQQPRASSGNGEARLNFGEPLMKNMSLRTMKIYLWTI
ICU11-5      1 MCNQTPLRSMALDSSGKQPEQQQQQQPRASSGNGEARLKTSENP----------------
ICU11        1 MCNQTPLRSMALDSSGKQPEQQQQQQPRASSGNGEARLKLRRTPNEEHEPENYEDLPLDY

ICU11-4     61 LLCSPLLSVTYLSSFSIPHESIKLVS----------------------------------
ICU11-6     61 VLLCSPLLSVTYLSSFSIPHESIKLVS---------------------------------
ICU11-7     61 VLLCSPLLSVTYLSSFSIPHESIKLVS---------------------------------
ICU11-5        ------------------------------------------------------------
ICU11       61 SPSLFTSLERYLPEQLLNSTRIDKASFMRDLLLRYSPDTERVRVLRHKEYRDKIMSSYQR
```

Supplementary Figure S1. Design and effects of the CRISPR/Cas9 mutagenesis of *ICU11*. **(A)** Details of the *ICU11* sgRNA1 target. The PAM sequence is shown in red. On-target mutation efficiency was calculated using the "Rule Set 2" scoring model, which provides values ranging from 0 to 100 (Doench et al., 2016). Possible off-target events are represented according to the number of mismatches [MM (number)]. **(B)** Electropherograms of the *ICU11* sgRNA1 target site in wild-type plants, and T_1 and T_2 transgenic plants. The gray, magenta, and orange shaded areas indicate chimeric, deletion, and insertion mutations, respectively. **(C)** Multiple amino acid sequence alignment of the predicted proteins translated from wild-type and CRISPR/Cas9 alleles showing that all the latter produce truncated proteins. Identical and similar residues are shaded in black and gray, respectively. Numbers indicate residue positions.

A Details of two putative off-targets of ICU11_sgRNA1

	ICU11_sgRNA1 off-target 1	ICU11_sgRNA1 off-target 2
Chromosome	1	3
Position	19132845	2280675
Strand	Forward	Complementary
Gene	Intergenic, between AT1G51590 (*MNS1*) and AT1G51600 (*GATA28*)	First exon of AT3G07170 (*IRP1*)
Mismatches	ICU11-T1: **GCGAGGCAAGATTGAAGCTTCGG**<br>Col-0: **GCGTGGAAATATTGAAACTTTGG**	ICU11-T1: **GCGAGGCAAGATTGAAGCTTCGG**<br>Col-0: **GCGAAGCAAGAATCAAACTTGGG**

B

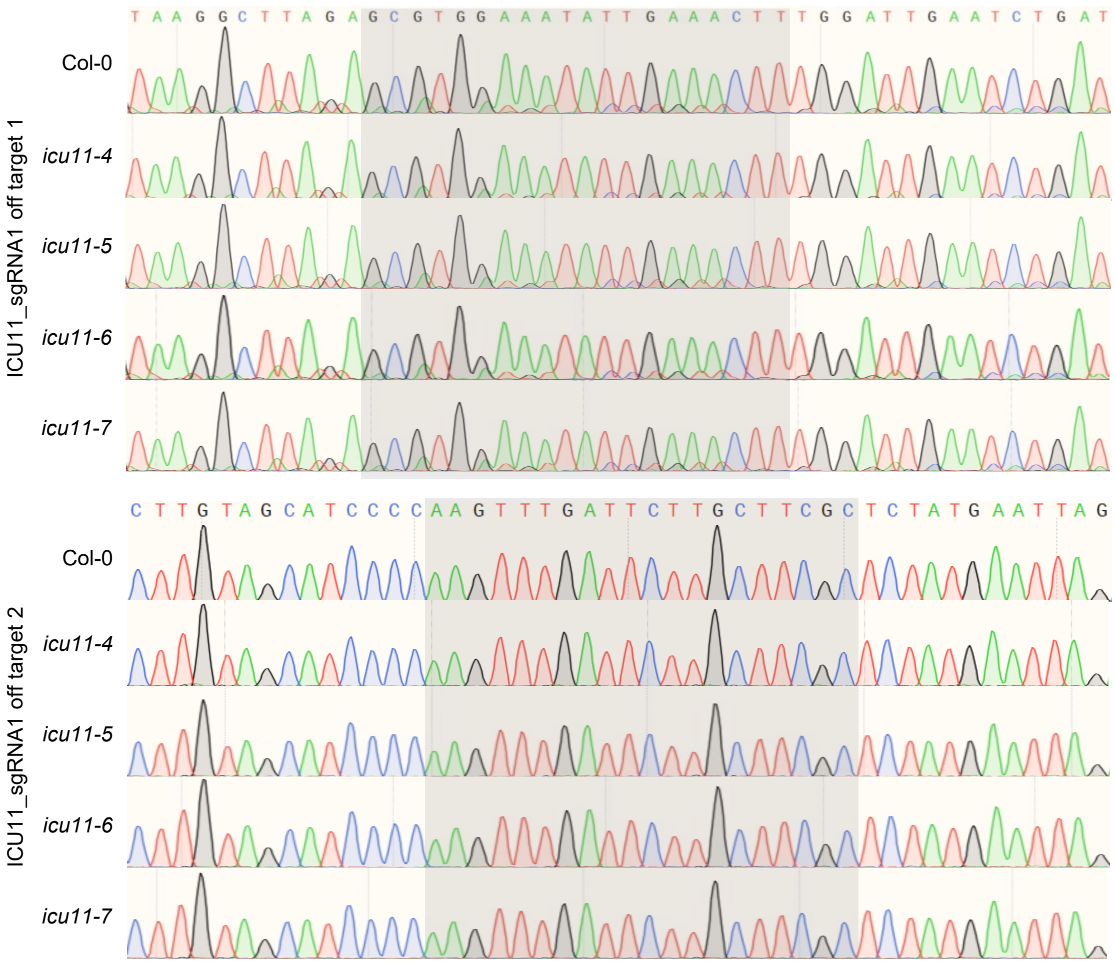

Supplementary Figure S2. Testing of two putative CRISPR/Cas9 off-targets in the new *icu11* mutant lines. **(A)** Off-targets were found using Cas-OFFinder, a bioinformatic tool developed by Bae et al. (2014). Mismatches are shown in red, with the first nucleotide of the PAM sequence (NGG) is in blue. **(B)** Sanger sequencing electropherograms of putative off-targets in T_3 mutant and wild-type plants. The gray shaded area corresponds to the putative off-target sequence.

Supplementary Table S1. Primer sets used in this work

Purpose	Oligonucleotide name(s)	Oligonucleotide sequences (5′ → 3′)	
		Forward primer (L or F)	Reverse primer (R)
Genotyping	ICU11-Off-target1_F/R	TGGTGGGTTTGGTTTGTCTC	CTCGGTCATTGGAGCAACTT
	ICU11-Off-target2_F/R	TGAGTCTGGAAGCAGGAAGG	AATGGGCAAATCAGAGAGTCC
	At1g22950_1F/R	ACCCTAACCTCTCAAACAAACCA	AGACTTTGTTAACCCAATCCGAC
	At1g22950_4F/R	CCTCTCAAACAAACCATCATCA	CGCTCAGTATCAGGGGAATATC
	SAIL_1215_B02_L/R	GAGCGATAACAGTGAGCTTGG	GACATTTTCAAACCATTCATGC
	SAIL_658_E12_L/R	AGAGGCAAGAGACGAAAAAGC	CCTTTGAGCCTGTAGCATCAG
	SAIL_621_G08_L/R	TGAGAGCGAAAGCTTTCATTC	AACAAATGACTGGAGCAGAGC
	gis-5_F/R	GAAGCAAGAACAGGTTTCTATG	AGCTAGTTACACTCGAGGATA
	icu2-1_F/R	TGTTGAAGGAGGTCAGTTATTCT	CACAAGTGTTTTGGATGACTGAA
	clf-2_F/R	ATGGCGTCAGAAGCTTCGCC	CTGGACCTCTCTCCTCCGC
	tfl2-2_F/R	TATCAGCGGTGATCGGTGTG	CGCCGTAATTCTCCCGGTAA
	ebs-1_F/R	TGAAGGTGTGAACAATGCAT	GAAAACTCGACCTGGTGTCG
	fas1-1_F/R	TGAGCTGTTCTTCTGCATCATG	ACTATGGTAGCTGTGAAGAGTG
	AT5G51230_1F/R	TGTAATGGTTCAGAGATCAATAGAA	GTCCGTGCAATCTTGAGAATG
	SALK_131712_L/R	CAGAAGAAGATCGTCCGAGTG	TGAACTTCCCCACTCTTCATG
	SALK_056440_L/R	TGGTCAGATGGGCTAGAATTG	AACGCGTTGCTGTAGAAACTC
	SAIL_826_A06_L/R	AGCAGCAGAAGAAGAAGCATG	TTTGGCCTACAAAGACACCAG
	SALK_021316_L/R	GAGCCGTCTCATCAAACTGAC	TTGCAGGAGCAAATATGGAAC
	SALK_150863_L/R	AGATCGCTTCCAGAGTTAGCC	TTGTCGCAAAAAGCAAAAGAG
	SAIL_223_F05_L/R	GGATCAGCCAAAAGGTTAAGG	TCATTCACTTTGCATCACTCG
	SAIL_809_E03_L/R	GCGTGTACCAGTTTCAAGGAG	TAAAGAGCCCAGTTGTGAAGC
	SALK_045303_L/R	CCAGTTAAGGACAGAACACCG	TCGTCTTTCGATCAAATCCAC
	SALK_022363_L/R	ATCAATGTGGCATCTAGTGGC	ACCCGCCTCTTCTTCATCTAC

Supplementary Table S1 (continued). Primer sets used in this work

Purpose	Oligonucleotide name(s)	Oligonucleotide sequences (5′ → 3′)	
		Forward primer (L or F)	Reverse primer (R)
Genotyping	SAIL_97_E06_L/R	CTTTCCCAGTTTTTACTGCCC	AATCACTCGCTTCTTCCACTG
	SALK_149002_L/R	AATGAAAGCATGCGGATACAC	TCCGTGTTGACTGGAAAGATC
	SALK_130607_L/R	TTTCTCTTGTCCGGTGAAATG	CCTGCAACAATCAGTGTGATG
	SAIL_240_H01_L/R	TTGAGATGAATCTGGAGACCG	AAACGACGACGTATTGGAGTG
	SALK_149692_L/R	TCTTGTGACAGGTGCAACTTG	AAACAAAGCTAGGCACAAGGC
	SALK_080380_L/R	AGGGAACATGTCATCCATGAG	AGGGAGAATCTGAGAACCTGC
	SALK_027726_L/R	ATGGTGTGCGAATCTATGACC	ACGGAGAGGAAAGCTCAAGAC
	LB1[1]	GCCTTTTCAGAAATGGATAAATAGCCTTGCTTCC	
	LbB1.3[2]	ATTTTGCCGATTTCGGAAC	
	Cas9_F/R	GCTTCATCAAGAGACAGCTGG	GGACTTGCCCTTTTCCACTTT
Cloning	ICU11_sgRNA1_F/R	ATTGCGAGGCAAGATTGAAGCTT	AAACAAGCTTCAATCTTGCCTCGC

[1,2]These primers were used for genotyping [1]SAIL and [2]SALK lines, and their sequences were taken from [1]Sessions et al. (2002) and [2]T-DNA Primer Design (http://signal.salk.edu/tdnaprimers.2.html).

SUPPLEMENTARY REFERENCES

Bae, S., Park, J., and Kim, J.S. (2014). Cas-OFFinder: a fast and versatile algorithm that searches for potential off-target sites of Cas9 RNA-guided endonucleases. *Bioinformatics* 30, 1473-1475. doi: 10.1093/bioinformatics/btu048

Cao, X., and Jacobsen, S.E. (2002). Role of the *Arabidopsis DRM* methyltransferases in de novo DNA methylation and gene silencing. *Curr. Biol.* 12, 1138-1144. doi: 10.1016/s0960-9822(02)00925-9

Chan, S.W., Henderson, I.R., Zhang, X., Shah, G., Chien, J.S., and Jacobsen, S.E. (2006). RNAi, DRD1, and histone methylation actively target developmentally important non-CG DNA methylation in *Arabidopsis*. *PLOS Genet.* 2, e83. doi: 10.1371/journal.pgen.0020083

Deng, W., Liu, C., Pei, Y., Deng, X., Niu, L., and Cao, X. (2007). Involvement of the histone acetyltransferase AtHAC1 in the regulation of flowering time via repression of *FLOWERING LOCUS C* in Arabidopsis. *Plant Physiol.* 143, 1660-1668. doi: 10.1104/pp.106.095521

Doench, J.G., Fusi, N., Sullender, M., Hegde, M., Vaimberg, E.W., Donovan, K.F., Smith, I., Tothova, Z., Wilen, C., Orchard, R., Virgin, H.W., Listgarten, J., and Root, D.E. (2016). Optimized sgRNA design to maximize activity and minimize off-target effects of CRISPR-Cas9. *Nat. Biotechnol.* 34, 184-191. doi: 10.1038/nbt.3437

Goll, M.G., Kirpekar, F., Maggert, K.A., Yoder, J.A., Hsieh, C.L., Zhang, X., Golic, K.G., Jacobsen, S.E., and Bestor, T.H. (2006). Methylation of $tRNA^{Asp}$ by the DNA methyltransferase homolog Dnmt2. *Science* 311, 395-398. doi: 10.1126/science.1120976

Jacob, Y., Feng, S., Leblanc, C.A., Bernatavichute, Y.V., Stroud, H., Cokus, S., Johnson, L.M., Pellegrini, M., Jacobsen, S.E., and Michaels, S.D. (2009). ATXR5 and ATXR6 are H3K27 monomethyltransferases required for chromatin structure and gene silencing. *Nat. Struct. Mol. Biol.* 16, 763-768. doi: 10.1038/nsmb.1611

Kim, J.S., Lim, J.Y., Shin, H., Kim, B.G., Yoo, S.D., Kim, W.T., and Huh, J.H. (2019). ROS1-dependent DNA demethylation is required for ABA-inducible *NIC3* expression. *Plant Physiol.* 179, 1810-1821. doi: 10.1104/pp.18.01471

Latrasse, D., Benhamed, M., Henry, Y., Domenichini, S., Kim, W., Zhou, D.X., and Delarue, M. (2008). The MYST histone acetyltransferases are essential for gametophyte development in Arabidopsis. *BMC Plant Biol.* 8, 121. doi: 10.1186/1471-2229-8-121

Lin, W., Sun, L., Huang, R.Z., Liang, W., Liu, X., He, H., Fukuda, H., He, X.Q., and Qian, W. (2020). Active DNA demethylation regulates tracheary element differentiation in *Arabidopsis*. *Sci. Adv.* 6, eaaz2963. doi: 10.1126/sciadv.aaz2963

Pien, S., Fleury, D., Mylne, J.S., Crevillen, P., Inzé, D., Avramova, Z., Dean, C., and Grossniklaus, U. (2008). ARABIDOPSIS TRITHORAX1 dynamically regulates *FLOWERING LOCUS C* activation via histone 3 lysine 4 trimethylation. *Plant Cell* 20, 580-588. doi: 10.1105/tpc.108.058172

Saze, H., Mittelsten Scheid, O., and Paszkowski, J. (2003). Maintenance of CpG methylation is essential for epigenetic inheritance during plant gametogenesis. *Nat. Genet.* 34, 65-69. doi: 10.1038/ng1138

Sessions, A., Burke, E., Presting, G., Aux, G., Mcelver, J., Patton, D., Dietrich, B., Ho, P., Bacwaden, J., Ko, C., Clarke, J.D., Cotton, D., Bullis, D., Snell, J., Miguel, T., Hutchison, D., Kimmerly, B., Mitzel, T., Katagiri, F., Glazebrook, J., Law, M., and Goff, S.A. (2002). A high-throughput Arabidopsis reverse genetics system. *Plant Cell* 14, 2985-2994. doi: 10.1105/tpc.004630

Woo, H.R., Dittmer, T.A., and Richards, E.J. (2008). Three SRA-domain methylcytosine-binding proteins cooperate to maintain global CpG methylation and epigenetic silencing in Arabidopsis. *PLOS Genet.* 4, e1000156. doi: 10.1371/journal.pgen.1000156

Yu, C.W., Chang, K.Y., and Wu, K. (2016). Genome-wide analysis of gene regulatory networks of the FVE-HDA6-FLD complex in *Arabidopsis*. *Front. Plant Sci.* 7, e555. doi: 10.3389/fpls.2016.00555

Yuan, L., Wang, D., Cao, L., Yu, N., Liu, K., Guo, Y., Gan, S., and Chen, L. (2020). Regulation of leaf longevity by DML3-mediated DNA demethylation. *Mol. Plant* 13, 1149-1161. doi: 10.1016/j.molp.2020.06.006

Yun, J.Y., Tamada, Y., Kang, Y.E., and Amasino, R.M. (2012). ARABIDOPSIS TRITHORAX-RELATED3/SET DOMAIN GROUP2 is required for the winter-annual habit of *Arabidopsis thaliana*. *Plant Cell Physiol.* 53, 834-846. doi: 10.1093/pcp/pcs021

Zheng, X., Pontes, O., Zhu, J., Miki, D., Zhang, F., Li, W.X., Iida, K., Kapoor, A., Pikaard, C.S., and Zhu, J.K. (2008). ROS3 is an RNA-binding protein required for DNA demethylation in *Arabidopsis*. *Nature* 455, 1259-1262. doi: 10.1038/nature07305
